# Emerging Roles of Microglia in Neuro-vascular Unit: Implications of Microglia-Neurons Interactions

**DOI:** 10.3389/fncel.2021.706025

**Published:** 2021-10-12

**Authors:** Zhe Ding, Shaohui Guo, Lihui Luo, Yueying Zheng, Shuyuan Gan, Xianhui Kang, Xiaomin Wu, Shengmei Zhu

**Affiliations:** ^1^Department of Anesthesiology, The First Affiliated Hospital, Zhejiang University School of Medicine, Hangzhou, China; ^2^Department of Anesthesiology, Zhejiang Provincial People’s Hospital, People’s Hospital of Hangzhou Medical College, Hangzhou, China

**Keywords:** microglia, microglia-neurons communication, synaptic plasticity, neuronal activity, NVU, ATP

## Abstract

Microglia, which serve as the defensive interface of the nervous system, are activated in many neurological diseases. Their role as immune responding cells has been extensively studied in the past few years. Recent studies have demonstrated that neuronal feedback can be shaped by the molecular signals received and sent by microglia. Altered neuronal activity or synaptic plasticity leads to the release of various communication messages from neurons, which in turn exert effects on microglia. Research on microglia-neuron communication has thus expanded from focusing only on neurons to the neurovascular unit (NVU). This approach can be used to explore the potential mechanism of neurovascular coupling across sophisticated receptor systems and signaling cascades in health and disease. However, it remains unclear how microglia-neuron communication happens in the brain. Here, we discuss the functional contribution of microglia to synapses, neuroimmune communication, and neuronal activity. Moreover, the current state of knowledge of bidirectional control mechanisms regarding interactions between neurons and microglia are reviewed, with a focus on purinergic regulatory systems including ATP-P_2_RY_12_R signaling, ATP-adenosine-A_1_Rs/A_2A_Rs, and the ATP-pannexin 1 hemichannel. This review aims to organize recent studies to highlight the multifunctional roles of microglia within the neural communication network in health and disease.

## Introduction

**Figure 1:**
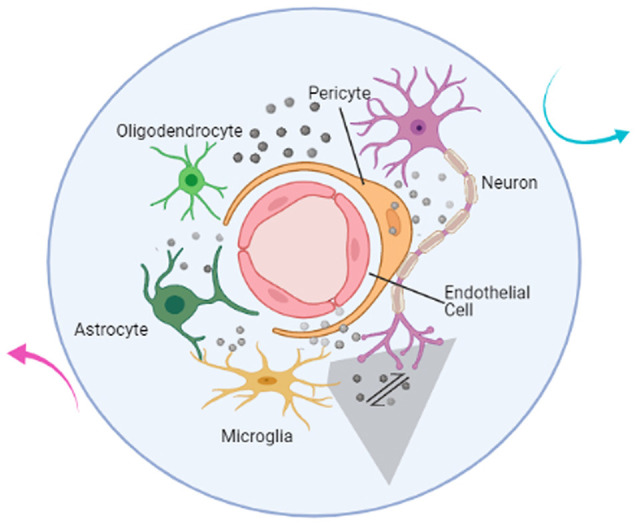
Illustration of the components of the neurovascular unit. The neurovascular unit (NVU) is composed of neurons (purple), microglia (yellow), astrocytes (dark green), oligodendrocytes (light green), endothelial cells (red), and pericytes (orange). Based on intimate anatomical relationships and chemical signals (gray sphere) secreted by oneself or others, these components establish an anatomical and functional whole (blue circle) that participates in physiological and pathological processes in the brain. The communication network also includes the interactions between NVU and the outside world (cyan and magenta arrow). The mutual communication and cooperation between microglia and neurons (gray shaded trapezoid) in the brain is the focus of this article and will be discussed below.

Research on central nervous system (CNS) disorders has largely concentrated on neurons; however, an increasing body of research suggests that a purely neurocentral focus is insufficient. All cell types in the brain—including neuronal, glial, and vascular components such as endothelia, pericytes, and vascular smooth muscular cells—should be examined in an integrated context (Muoio et al., 2014). Together, these components are termed the neurovascular unit (NVU; Figure 1), which plays an important role in brain function and disease through cell–cell signaling (Iadecola, 2017). The NVU is reported to control blood-brain barrier (BBB) permeability and cerebral blood flow (CBF) and to regulate the neuronal ‘milieu’ (Zlokovic, 2011). Signaling perturbations within the NVU comprise potential mechanisms for neuronal dysfunction and injury (Sweeney et al., 2016; Yu et al., 2020). Recent studies have shown that the NVU contributes to both stroke and neurodegenerative diseases (Cai et al., 2017; Giaume et al., 2021; Minhas et al., 2021). In light of such findings, increasing research efforts have focused on the NVU as a therapeutic target (Quaegebeur et al., 2011).

Microglia perform an immune surveillance function through highly motile protrusions and ramified processes that sense their environment during their “resting” state or after activation *in vivo* (Nimmerjahn et al., 2005). Notably, microglia do not patrol in an aimless manner; rather, microglial surveillance is associated with neuronal activity and synaptic plasticity (Nayak et al., 2014). Emerging evidence suggests that microglia contribute to the development, maturation, and aging of the brain (Liu et al., 2020; Verkhratsky et al., 2021) and are recognized to express many risk genes for CNS disorders, including genes associated with Alzheimer’s disease (AD), Parkinson’s disease (PD), schizophrenia, autism, and multiple sclerosis (MS; Kékesi et al., 2019; Prinz et al., 2019). Neuron-microglia signaling can be detected under physiological and pathological conditions in the brain (Cserép et al., 2020, 2021). The interactions between microglia and neurons establish complex regulatory loops that involve either the establishment of neural networks and maintenance of neural circuits in health or the development of neurological disorders in disease (Cserép et al., 2021).

Microglia, as a component of the NVU, play significant roles in neuroinflammation and innate immunity (Lehnardt, 2010; Joost et al., 2019). Not only do microglia-derived mediators participate in inflammation and immune-related responses they also serve as important messengers in cell-cell signaling between neuronal and glial cells. In this review, we discuss the evidence of the contribution of microglia to synapses, neuroimmune communication, and neuronal activity. In particular, we focus on bidirectional interactions between microglia and neurons that depend on soluble factors and intercellular signaling pathways, with the ultimate aim of better understanding the recently recognized functional roles of microglial actions in synaptic function, neuroimmune responses, and regulation of neural activity.

## A Synaptic Role for Microglia

During postnatal development of the brain, microglia play major roles in the rapid elimination of dying neurons and synaptic structures (Tremblay et al., 2010; Ayata et al., 2018), synaptic pruning (Paolicelli et al., 2011; Schafer et al., 2012) and promoting the survival of cortical neurons (Ueno et al., 2013). Maternal immune activation (MIA), which is induced by injecting pregnant mice with polycytidylic acid (poly I:C), has been shown to have a significant impact on the pre-microglia (from embryonic day 14 to a few weeks after birth) of newborn offspring. As this impact on pre-microglia is influencing neurogenesis and synaptic pruning, it explains the later occurrence of behavioral disorders when the offspring are adults (Matcovitch-Natan et al., 2016). Emerging evidence suggests that microglia regulate activity-dependent structural plasticity and promote memory consolidation by locally clearing the extracellular matrix (ECM). Furthermore, cytokine interleukin-33 (IL-33), which is expressed by hippocampal neurons, significantly upregulates ECM protease genes (namely *Adamts4* and *Mmp14*), thereby promoting microglial phagocytosis and engulfment of aggrecan around parvalbumin+ interneurons in the CA1 hippocampal subregion (Nguyen et al., 2020). Moreover, this mechanism is demonstrated in both young mouse (3 months) and old mouse (18 months) under physiological conditions.

The release of immunological mediators from microglia has also been shown to influence synaptic function. Beattie et al. (2002) reported that tumor-necrosis factor-alpha (TNFα) significantly increases the mean miniature excitatory postsynaptic currents (mEPSCs) frequency and promotes the maintenance of synaptic strength, as indicated by mEPSCs at excitatory synapses that call for the continual presence of TNFα, which enhances synaptic efficacy by increasing surface expression of AMPA receptors. Since microglia are a source of TNFα, they potentiate glutamate-mediated neurotoxicity or participate in synaptic connectivity in an indirect way (Stellwagen and Malenka, 2006; Olmos and Lladó, 2014). Interleukin 1β (IL-1β) has been shown to impair long-term potentiation (LTP) directly at the synapse, which could explain why cognitive impairment is worse in aged hippocampal synapses (Prieto et al., 2015). Aberrant expression of IL-1β also results in damage to hippocampus-dependent memory (Patterson, 2015). It is interesting that either TNFα or IL-1β plays a role in sleep regulation by changing neuromodulator/neurotransmitter receptor expression, resulting in altering neuron sensitivity (Krueger et al., 2011). Brain-derived neurotrophic factor (BDNF), which is reported to influence synaptic plasticity, learning, and memory formation (Parkhurst et al., 2013; Leal et al., 2014), also appears to be a crucial signaling molecule in microglia-neuron communication. Preventing BDNF release from microglia has been shown to reverse allodynia and anion shift, and may thus provide a new therapeutic strategy for treating neuropathic pain (Coull et al., 2005). Interleukin 10 (IL-10), which acts on IL-10 receptors expressed on hippocampal neurons, plays a role in the induction of synaptic formation, including dendritic spines and excitatory and inhibitory synapses (Lim et al., 2013). It has been shown that IL-10 can facilitate LTP in the CA1 region of the hippocampus and increase synaptic strength, based on observations that the presynaptic fiber volley field excitatory postsynaptic potential (FV-fEPSP) slope increases after IL-10 treatment, thereby promoting synaptic plasticity (Nenov et al., 2019).

These soluble factors, which have been extensively studied in the context of neuroinflammation (Perry and Holmes, 2014), exert their effects on synaptic function as discussed above. The released cytokines act on neurons in a flexible way and are not limited by distance. Studies conducted in recent years have shown that the administration of cytokines, including TNFα and IL-1β, results in significantly increased astrogliosis at the brain injury site in neonatal mouse (Balasingam et al., 1994). Liddelow et al. (2017) reported that the release of IL-1α, TNF, and C1q from microglia is necessary and sufficient to induce a subtype of reactive astrocytes (termed A1 astrocytes), leading to impairment of neuronal survival, outgrowth, synaptogenesis, and phagocytosis and the death of neurons and oligodendrocytes. In response to cytokines, astrocytes can produce IL-1, IL-4, IL-6, IL-10, IL-12, TNF-α, and interferon (IFN) to act on microglia (Benveniste, 1998). Taken together, these studies have shown that soluble factors contribute to the establishment of the interplay between different cell types within the NVU. The influence of microglia on neuronal networks appears to be sophisticated since interactions within the NVU cannot be ignored. Thus, the net effects must be taken into consideration. In light of this knowledge, the application should be carefully considered, since the ultimate effects of immunological mediators appear to depend on time, concentration, and environmental milieu.

## Neuroimmune Communication with Microglia

Microglia are the resident myeloid cells of the brain (Nayak et al., 2014). Equipped with specialized pattern recognition receptors (PRRs) that identify pathogen-associated molecular patterns (PAMPs) and host-derived danger-associated molecular patterns (DAMPs) in microorganisms, microglia play a key role in the neuroimmune system by quickly inducing a fine-tuned inflammatory response (Scheiblich et al., 2020). Activation of microglia that play a role in innate immune function is pivotal in neuroinflammation. In addition to their influences on synaptic plasticity, the release of cytokines and chemokines further amplifies the inflammatory process, which has been well documented in previous studies (Nayak et al., 2014; Prinz et al., 2019).

The nervous system is involved in regulating immunity and inflammation, whereas immune dysregulation and inflammation also affect brain function in disease (Pavlov et al., 2018). For instance, neural circuits may promote immunosuppression after injury or stroke by altering microglia function (Pavlov and Tracey, 2017). Notably, MIA, which involves alterations in the number and state of activated microglia, is closely associated with early disruptions in neurodevelopment and may result in later neuropsychiatric disorders in offspring, including anxiety-like or depression-like behavior, sensorimotor deficits, social deficits, and repetitive behaviors. Gumusoglu and Stevens (2019) proposed that the most common outcomes of maternal immune perturbation are elevations in the proinflammatory cytokines IL-6 and IL-1β, resulting in alterations in synapse formation and function and driving neural progenitors from a neurogenic to gliogenic fate. Brown et al. (2017) revealed that exposure to intrauterine inflammation altered metabolic profiles, including amino acid metabolism, purine metabolism, and lipid metabolism, in the amniotic fluid and fetal and neonatal brain of exposed offspring. Interestingly, the changes were sex-specific and persisted at a later time point (48 h vs. 6 h after intrauterine inflammation; Brown et al., 2017). Furthermore, metabolic pathways disturbances, including mitochondrial function, arborization, and synapse formation, have been observed in the developmental interneurons of individuals with schizophrenia (SCZ) co-cultured with activated microglia. Intriguingly, SCZ cortical interneurons (cINs) show prolonged compromised metabolic pathways after removal of the activated microglia, which indicates an interaction between the genetic background of SCZ donors and the inflammatory environment provided by activated microglia (Park et al., 2020). It has been suggested that neuroinflammation mediated by microglia specifically acting on cINs has long–term effects during brain development. Notably, the microglial gene expression profiles of the offspring of mothers administered poly I:C were realigned with the normal microglial phenotype at adulthood, indicating that transient perturbation in the early stage of microglia development (such as the pre-microglia stage) may have far-reaching implications on the brain at adulthood (Matcovitch-Natan et al., 2016).

Recent studies suggest that any assessment of the impact of microglia-mediated immune responses on neurons should consider differences between short-term and long-term effects. It can be concluded that the activation of microglia following immune activation induces inflammation of the brain, which has a profound impact on neurodevelopment. Although microglia are primary initiators and effectors of neuroinflammation (Thurgur and Pinteaux, 2019), the pathological process includes the contributions of various cell types. It has been established that astrocytes along with microglia participate in the amplification of inflammation signals, which in turn may cause apoptosis of oligodendroglioma cancer cells (Liddelow et al., 2020). More precisely, microglia are essential for triggering the standard immune response of microglia with astrocytes, as confirmed by studies revealing that astrocytes show no response to pathogens/damage in the absence of microglia (Liu et al., 2021). At the same time, neuroimmune communication also occurs between microglia and endothelial cells, where microglia play a role in maintaining the integrity of the BBB and thus the influx of blood-derived molecules into the brain (Muoio et al., 2014).

In addition, recent studies have uncovered that neuroimmune interactions are also important regulators of physiology (Huh and Veiga-Fernandes, 2020). For example, microglia work to maintain homeostasis in the brain, not only to resolve infections (Norris and Kipnis, 2019). Pavlov and Tracey (2017) revealed neural reflex regulation of immunity, finding that neural circuits are triggered by and control inflammation. Sensory neurons are capable of monitoring the immune state in the periphery and interacting with immune cells, which are defined as neuroimmune cell units. The communicating immune signals, which then activate the CNS, can derive from any major body organ including skin, lung, and intestines (reviewed in Huh and Veiga-Fernandes, 2020). Ultimately, the brain integrates neuro-immune communication in order to maintain a steady-state.

## Microglia-Neurons Communication

New evidence suggests that exacerbated activation of microglia can promote microglia-mediated neuronal degeneration (Zhao et al., 2018). It has been shown that loss of striatal microglia triggers seizures *via* activation of cortical and hippocampal neurons in mice (Badimon et al., 2020). Moreover, similar to inhibitory neurons, microglia can sense neuronal activation and then lower the activation of dopaminergic neurons to achieve negative feedback control of neuronal activity (Badimon et al., 2020). In addition, neuronal excitation affects the process extension and motility of microglia (Liu et al., 2019; Stowell et al., 2019). Overall, these studies support reciprocal connections between microglia and neurons. Understanding the molecular mechanisms that underlie these bidirectional interactions will be necessary to achieve an integrated view of microglia-neuron communication systems, thereby enabling real insight into the importance of these communication systems in the control of brain function.

### ATP-P_2_RY_12_ Signaling Is Essential for Microglia Neuron Communication

**Figure 2:**
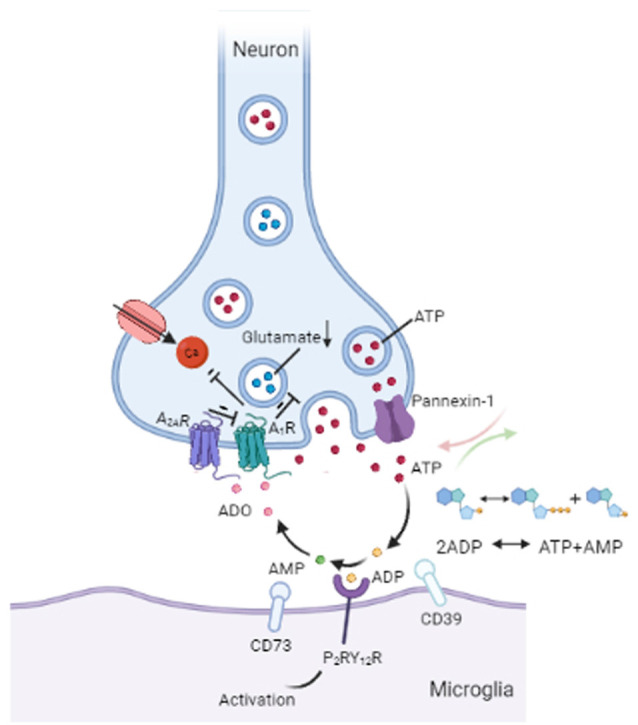
Communication between microglia and neurons. ATP can be secreted from neurons through vesicles or the pannexin-1 channel. Released ATP (red) is converted into ADP (yellow) by the microglial enzyme CD39. ADP acts on P_2_RY_12_ receptors to induce activation of P_2_RY_12_ signaling, which attracts microglial processes to synaptic connections. CD39 also converts ADP into AMP (green). Next, AMP is converted into ADO (pink) by the enzyme CD73 on microglia. ADO suppresses neuronal activity by acting on A_1_Rs by decreasing glutamate (blue) release and inhibiting calcium (Ca^2+^) channels. In addition, A_2A_Rs can weaken the inhibitory effect of A1Rs on glutamate release. ATP = adenosine triphosphate, ADP = adenosine diphosphate, AMP = adenosine monophosphate, ADO = adenosine, A_1_R = A_1_ receptor, A_2A_R = A_2A_ receptor.

Purinergic signaling plays a central role in microglia-neuron interactions, of which ATP is recognized as an efferent neurotransmitter in the CNS (Burnstock, 2006; Burnstock et al., 2011). ATP, which is mediated by G-protein-coupled P_2_ receptors, acts as an activity-dependent signal under physiological conditions or as a danger signal when dysfunction or damage occurs in the brain (Agostinho et al., 2020). There is evidence of the vesicular release of ATP from neurons (Pankratov et al., 2007; Larsson et al., 2012) and from astrocytes (Darby et al., 2003; Bowser and Khakh, 2007). ATP released by astrocytes regulates microglial branch dynamics in the intact brain and chemotactic responses of activated microglia toward the local injury site in the brain (Davalos et al., 2005). In neuron-microglia interactions, ATP supports communication from neurons to microglia *via* P_2_RY_12_ signaling activation (Badimon et al., 2020; Figure 2). In general, ATP results in a decrease in neuronal activity both in normal (Li et al., 2012) and in pathological conditions (Cserép et al., 2020). Microglial P_2_RY_12_ receptors are exclusively expressed by microglia and are viewed as an indispensable component of microglia-neuron junctions (Eyo et al., 2014; Cserép et al., 2020). 3D electron tomography showed that P_2_RY_12_ receptor density was negatively correlated with the distance between microglial and neuronal membranes within the junctions. Meanwhile, *in vivo* 2P imaging further confirmed that microglial process recruitment to somatic junctions is linked to the metabolic activity of neuronal mitochondria through a P_2_RY_12_ receptor–dependent mechanism (Cserép et al., 2020). Emerging evidence suggests that P_2_RY_12_Rs are involved in chemotaxis and the motility of microglia (Dissing-Olesen et al., 2014) as well as in microglia activation in the trigeminal nucleus caudalis (Jing et al., 2019), neuropathic pain (Tozaki-Saitoh et al., 2008; Gu et al., 2016), epilepticus (Avignone et al., 2008; Milior et al., 2020), and stroke (Kluge et al., 2017; Li et al., 2020). Thus, ATP-P_2_RY_12_R signaling responses between microglia and neurons appear to contribute to an important loop in neural crosstalk (Figure 2).

### ATP-Adenosine-A_1_Rs/A_2A_Rs Are Essential for Microglia Neuron Communication

Following ectoenzymatic breakdown of extracellular ATP, adenosine is produced and binds to A_1_ or A_2A_ receptors in the brain, thereby regulating nerve activity and transmitter release (Fredholm et al., 2005). It has been demonstrated that adenosine (ADO) lowers neuronal excitability by acting on A_1_Rs and is a prominent physiological mediator of sleep homeostasis (Ribeiro et al., 2002). Recent studies have revealed that cholinergic and glutamatergic neurons show highly correlated activity with changes in adenosine concentration, with glutamatergic neurons contributing much more to adenosine increases in the mouse basal forebrain (BF; Peng et al., 2020). A_1_Rs and A_2A_Rs are widely abundant in the brain, particularly in glutamatergic synapses (Yoon and Rothman, 1991; Rosin et al., 1998; Rebola et al., 2005). As a synaptic neuromodulator curtailing excitatory synaptic transmission, A_1_Rs-mediated inhibition can fully block glutamatergic transmission (Coelho et al., 2006; Rodrigues et al., 2008) by inhibiting adenylate cyclase (AC), increasing potassium (K^+^) conductance, and inhibiting presynaptic calcium (Ca^2+^) channels (Benarroch, 2008). A_2A_Rs are activated by ATP-derived adenosine upon increased synaptic activity to act on synaptic plasticity (d’Alcantara et al., 2001; Augusto et al., 2013; Kerkhofs et al., 2018). In contrast, presynaptic A_2A_Rs may form heteromeric receptor complexes with presynaptic A_1_Rs (Benarroch, 2008), which antagonizes the inhibitory effect of presynaptic A_1_Rs on glutamate release from axon terminals in the striatum, cerebral cortex, and brainstem (Ribeiro et al., 2002; Boison, 2006). In addition to ATP-P_2_RY_12_R signaling as described above, a recent study demonstrated that ATP-adenosine-A_1_Rs signaling in mice suppresses D1 neuronal activity in the striatum, which could be regarded as a novel mechanism for communication between microglia and neurons (Badimon et al., 2020). Metabolic stress, such as ischemia, seizures, or trauma, may result in the upregulation of extracellular adenosine (Benarroch, 2008). The release of adenosine from parallel fibers has also been reported in the rat cerebellum. Moreover, activity-dependent adenosine release is known to regulate cerebellar circuit output through feedback inhibition of parallel fiber-Purkinje cell transmission (Wall and Dale, 2007). Much research consideration has surrounded adenosine as an endogenous neuromodulator in the CNS (Benarroch, 2008). Such studies have revealed roles for adenosine as well as adenosine receptors, greatly adding to our understanding while simultaneously expanding the complexity of the signaling system’s mechanisms of action.

### ATP-Pannexin 1 Hemichannel Are Essential for Microglia Neuron Communication

Pannexin 1 forms intercellular hemichannels and plays multiple roles in channel-mediated ATP release (Dahl and Locovei, 2006; Bhat and Sajjad, 2021). As nonjunctional membrane channels, pannexin 1 is abundantly expressed in the brain, including the hippocampus, olfactory bulb, cortex, and cerebellum (Bruzzone et al., 2003; Ray et al., 2005). These hemichannels can be activated by extracellular Ca^2+^ under physiological conditions (Barbe et al., 2006), and alterations in intracellular Ca^2+^ levels also open pannexin 1 channels (Giaume et al., 2021). Pannexin 1 channel-mediated ATP release has been shown to contribute to cell communication *in vivo* (Giaume et al., 2021; Figure 2). Reciprocal regulation between microglia and neurons involves pannexin-1 hemichannels in neurons and ATP/P_2_ receptors in microglia, and so intraneuronal calcium plays a functional role in neuronal activity-induced microglia-neuron contact (Li et al., 2012). When pannexin 1 hemichannels are inhibited by trovafloxacin, both ATP release and migration of microglia are significantly repressed, resulting in the reduction of pro-inflammatory cytokines (Garg et al., 2018). Probenecid, which is an inhibitor of the pannexin 1 hemichannel, has been demonstrated to attenuate cognitive impairment after cecal ligation and puncture (CLP)-induced sepsis in mice by inhibiting pannexin 1-dependent ATP release in the hippocampus (Zhang et al., 2019). In addition, recent findings have proposed a role for pannexin-1 hemichannels in the suppression of glutamate release from hippocampal CA1 pyramidal neurons in male rat or mouse brains (Bialecki et al., 2020). In mice with pannexin-1 channels blocked or genetically deleted, the onset of seizures is reduced in neocortical slices *in vitro* (seizure-like events) and the hippocampal CA3 region *in vivo*, indicating a pivotal role of pannexin-1 in maintaining neuronal hyperexcitability (Aquilino et al., 2020). Similarly, the activation of pannexin-1 hemichannels in postoperative tissue samples from patients with epilepsy promotes seizure generation and maintenance through ATP signaling *ex vivo* (Dossi et al., 2018). Importantly, the ATP, glutamate, and other metabolites released from stimulated pannexin-1 hemichannels can go on participating in cell-to-cell communication in the brain (Aquilino et al., 2019).

Collectively, microglia are tightly regulated by the brain microenvironment and controlled by a sophisticated system of receptors and signaling cascades (Figure 2). However, there are some open questions worth discussing. For instance, ATP itself can act presynaptically, rather than solely postsynaptically, in the CNS (Cunha and Ribeiro, 2000). Despite a series of articles on the mechanisms of purinergic regulatory systems during microglia-neuron communication (Phillis and Wu, 1981), the balance between the effects of different signaling is yet to be considered. Besides acting on microglia and neurons, both ATP and adenosine are recognized to play a role in astrocyte proliferation and the formation of reactive astrocytes (Fumagalli et al., 2003). Similar to microglia, astrocytes are capable of releasing ATP and adenosine, which are then involved in cell–cell communication (Stout et al., 2002; Agostinho et al., 2020). ATP can be released along with neurotransmitters from nerve terminals, acting either as a neurotransmitter in central synapses or as a neuromodulator (Agostinho et al., 2020). Hence, the proposal of the NVU was quite significant and has inspired researchers to explore the signaling mechanisms spatially and temporally.

## Conclusions and Perspectives

In this review, we have brought together a series of articles exploring the roles of microglia, including their contributions to synapses, neuroimmune communication, and neuronal activity. In recent years, the development of imaging, genetics, and sequencing has made it possible to understand the real story of the complex and fascinating roles of microglia in health and disease. As a result, microglia manipulation has been proposed as a novel therapeutic method for modulating activity in various neurological diseases (Klawonn et al., 2021; Minhas et al., 2021). In the context of the neuroimmune roles of microglia, targeting microglia for immunotherapy in neurological disorders is aimed to maintain homeostasis of the brain by controlling neuroinflammation. However, the appropriate way to use microglia in immunotherapy remains unclear since they display both beneficial and detrimental roles. To gain deeper insight into the microglial shift from being protective to pathogenic, the molecular mechanisms involved warrant extensive study.

The overlapping results across MIA studies (reviewed in Gumusoglu and Stevens, 2019) inspire us that we can evaluate the psychiatric risk and disease etiologies by clinically testing the immune milieu of offspring. However, as discussed above, the transient perturbation in microglia development could have a profound impact on neurodevelopment. It still requires more efforts to prevent the occurrence of neuropsychiatric disorders in offspring. Moreover, inhibiting specific molecular pathways that mediate neuronal microglial communication is also a promising therapeutic approach, such as the application of probenecid in cerebral dysfunction of sepsis (Zhang et al., 2019). Novel research has reported that neuron-derived IL-33 (Nguyen et al., 2020) and astrocyte-derived IL-3 (McAlpine et al., 2021) as a key mediator of neuron-microglia crosstalk and astrocyte-microglia crosstalk, respectively, are associated with memory consolidation. It provides a new strategy for the future treatments of age-related memory decline (IL-33) and Alzheimer’s disease (IL-3). In addition, eliminating about 80% microglia in the 5xfAD mouse model of AD by blocking the CSF1 receptor is able to reverse deficits in contextual memory *via* preventing dendritic spine loss and neuronal loss, despite the disease being at a late stage (Spangenberg et al., 2016).

Another challenge is that bulk interventions using microglia could veil the true physical function of microglia since the overall outcome would be determined by microglia-mediated changes at different temporal and spatial scales (Cserép et al., 2021). The different effects could also be explained by the multifunctional roles of microglia and their communication within the NVU. Therefore, future studies should consider the molecular anatomy and functional heterogeneity of microglial processes and compartment-specific actions by microglia.

## Author Contributions

ZD and SG wrote this manuscript. LL, YZ, SG, and XK revised the manuscript. SZ and XW designed the general idea. All authors contributed to the article and approved the submitted version.

## Conflict of Interest

The authors declare that the research was conducted in the absence of any commercial or financial relationships that could be construed as a potential conflict of interest.

## Publisher’s Note

All claims expressed in this article are solely those of the authors and do not necessarily represent those of their affiliated organizations, or those of the publisher, the editors and the reviewers. Any product that may be evaluated in this article, or claim that may be made by its manufacturer, is not guaranteed or endorsed by the publisher.
